# Multi-Frequent Band Collaborative EEG Emotion Classification Method Based on Optimal Projection and Shared Dictionary Learning

**DOI:** 10.3389/fnagi.2022.848511

**Published:** 2022-02-17

**Authors:** Jiaqun Zhu, Zongxuan Shen, Tongguang Ni

**Affiliations:** School of Computer Science and Artificial Intelligence, Changzhou University, Changzhou, China

**Keywords:** cognitive computing, **E**EG-based emotion classification, multi-frequency band EEG signals, subspace learning, dictionary learning

## Abstract

Affective computing is concerned with simulating people’s psychological cognitive processes, of which emotion classification is an important part. Electroencephalogram (EEG), as an electrophysiological indicator capable of recording brain activity, is portable and non-invasive. It has emerged as an essential measurement method in the study of emotion classification. EEG signals are typically split into different frequency bands based on rhythmic characteristics. Most of machine learning methods combine multiple frequency band features into a single feature vector. This strategy is incapable of utilizing the complementary and consistent information of each frequency band effectively. It does not always achieve the satisfactory results. To obtain the sparse and consistent representation of the multi-frequency band EEG signals for emotion classification, this paper propose a multi-frequent band collaborative classification method based on optimal projection and shared dictionary learning (called MBCC). The joint learning model of dictionary learning and subspace learning is introduced in this method. MBCC maps multi-frequent band data into the subspaces of the same dimension using projection matrices, which are composed of a common shared component and a band-specific component. This projection method can not only make full use of the relevant information across multiple frequency bands, but it can also maintain consistency across each frequency band. Based on dictionary learning, the subspace learns the correlation between frequency bands using Fisher criterion and principal component analysis (PCA)-like regularization term, resulting in a strong discriminative model. The objective function of MBCC is solved by an iterative optimization algorithm. Experiment results on public datasets SEED and DEAP verify the effectiveness of the proposed method.

## Introduction

Affective computing focuses on how to actively learn, reason, and perceive the surrounding world, as well as realize a certain level of brain-inspired cognitive intelligence by simulating people’s psychological cognitive processes (Aranha et al., 2019; Samsonovich, 2020). Researchers in psychology and neurobiology investigate the changes and relationships in the human physiological systems that occur during various emotional states and activities (Li et al., 2020). More and more evidences show that with the progress of neuroscience research, there is a connection between human emotional activity and the activity of specific areas of the brain, especially the cerebral cortex and central nervous system. For example, the amygdale is associated with emotions like fear and anxiety in the limbic system of the brain. Anger can activate the left frontal lobe of the brain (Davis and Whalen, 2001). Researchers have also studied the relationship between certain diseases and emotional activities, such as cancer, cardiovascular disease, and depression (Zhao et al., 2018). Wirkner et al. (2017) and Guil et al. (2020) studied the impact of emotional activity on the progression of breast cancer patients. Nurillaeva and Abdumalikova (2021) studied the pathways of communication between the heart and the brain, as well as the relationship between heart rhythm and cognitive and emotional functions. According to the study of Gianaros et al. (2014), there is a link between affective regulation and cardiovascular disease. The author discussed how intense emotional activity and the immune system interact, and how these close interactions affect the treatment of rheumatic cardiovascular disease. Tennant and McLean (2001) associated mood disorders such as anxiety, depression, and anger with coronary heart disease. Authors classified mood disorders as an important risk factor for coronary heart disease, and concluded that mood disorders are frequently associated with coronary heart disease events. Klatzkin et al. (2021) studied the food intake of emotional dieters during various emotional and stress responses. Researchers are also interested in the impact of emotional activities in the business field. According to research on the effect of emotion on commercial advertising, advertisements with emotional expression and influence are easier for consumers to remember, and publicity images with emotional color can influence consumers’ access behavior (Shareef et al., 2018). It is clear that research on human emotional activities is important not only in the study and understanding of humanity, but also in medical health and commercial activities. As a result, the study of human emotions, including emotional activity intervention, can be regarded as scientific and practical.

Electroencephalogram activities are closely related to people’s psychological attention consciousness and emotional experience. An emotional EEG signal is a physiological electrical signal collected by the human brain in a specific emotional state. EEG signals, as a window into brain thinking activities, cognitive processes, and mental states, are an important technical means for studying brain function and its neural mechanism. Wearable devices placed on the top of the head collect emotional EEG signals. The acquisition electrode’s placement position is typically determined using the international standard 10–20 and other systems. Researchers in the field of artificial intelligence study the relationship between emotional activities caused by internal and external stimuli and the content of stimuli. Machine learning technology in artificial intelligence is widely used in EEG signals-based emotion classification. For example, Liu et al. (2020) developed a multi-level features guided capsule network to describe the internal relationship of multiple EEG signal channels. The advantage of this model is that different levels of feature mapping are integrated during the process of forming the primary capsule, which can improve feature representation ability. Zhong et al. (2020) proposed a regularized graph neural network to mine both local and global relationships between various EEG channels. This method can alleviate the problem of time dependence in emotional process. Ni et al. (2021) developed a domain adaptation sparse representation classification model to alleviate the problem of insufficient training data in the new scene. This method employed the discriminative knowledge of historical data or related data to aid in establishing the classification model of the current scene.

According to intra-band correlation with a distinct psychological state, the EEG signals can be split into five frequency bands. Different frequency band EEG signals reflect the different states of brain state. Table 1 briefly describes the information of five frequent bands of EEG signals (Gu et al., 2021a; Shen et al., 2021). Many scholars have studied EEG signals in different frequency bands. Mohammadi et al. (2017) used wavelet transform to decompose EEG signals into five sub-band signals, then extracted entropy and energy features from each sub-band signal and sent them to support vector machine and *k*-nearest neighbor, respectively. Li and Lu (2009) proposed a frequency band search method to find the best frequency band for emotion classification. According to their findings, the gamma frequency band is appropriate for EEG-based emotion classification using still images as stimuli. Zheng and Lu (2015) built a Multi-frequent band emotion recognition classifier using deep neural networks. This study had shown that the beta and gamma bands contained more discriminative information for emotion classification. Li et al. (2018) used the hierarchical deep learning model to train numerous classifiers on EEG signals. They verified that high-frequency bands played the most important role in emotion classification. Yang et al. (2018) developed a 3D representation of signal segment to extract representative features on bands. They integrated multiple frequency bands and used the constructed 3D signal cube as model input. Li et al. (2019) developed a sparse linear regression model using the technologies of graph regularization and sparse regularization. The authors compared the effects of different frequency band signals in emotion recognition on various EEG datasets.

**TABLE 1:** The basic information of five frequent bands of EEG signals.

Patterns	Frequency	Brain state
Delta (δ)	1–3 Hz	Slowest “sleep waves”
Theta (θ)	4–7 Hz	Light meditation and sleeping
Alpha (α)	8–13 Hz	Closing the eyes, relaxation
Beta (β)	14–30 Hz	Waking consciousness and reasoning waves
Gamma (γ)	30–100 Hz	Sensory and high-level information processing

Because there are internal relationships and differences between different frequency bands, a new learning method is required to make full use of the information in multi-frequency band data. Despite extensive research on the use of different frequency bands of EEG signals for emotion recognition, one traditional strategy is to directly concatenate features from multi-frequent bands in high dimensional space and consider this single feature vector as the model’s input. Obviously, this strategy does not account for the complementarity and consistency of the data in each frequency band.

Our previous work named as optimized projection and Fisher discriminative dictionary learning (OPFDDL) (Gu et al., 2021a) extracted multi-frequent band EEG features in the optimal sparse representation subspace, and adopted the Fisher discrimination criterion to build a discriminative classifier. This method did not directly concatenate the features of each frequency band, but regarded each band signal as an independent feature. It incorporated the band-correlation knowledge into a dictionary learning model by learning independent projection matrices for each frequency band signal. Inspired of this work, we further use multi-frequent band shared information to exploit the intrinsic knowledge of EEG signals and achieve correlation modeling of multiple band data. Thus, in this study we propose a multi-frequent band collaborative EEG emotion classification method based on optimal projection and shared dictionary learning (MBCC). We construct a projection matrix for each frequency band. The projection matrix is composed of a common shared matrix (called shared component) and a frequency band-specific matrix (called specific component). The shared matrix well reflects the relationship between frequency bands. The EEG signal of each frequency band is projected to the subspace through the projection matrix, and the dictionary shared by each frequency band is learned in the subspace. The corresponding sparse representation is then obtained from the new data features using dictionary learning. According to Fisher’s criterion, the MBCC method ensures that the coding reconstruction errors of the same class are as small as possible, while the coding reconstruction errors of different classes are as large as possible. Considering the information available in the original domain should not be lost in the projection space, we provide a regularization term similar to principal component analysis (PCA) that can retain discriminative knowledge to improve the discrimination ability of the model. An efficient alternating iterative optimization algorithm is designed to solve the proposed model. The experiment yielded good classification results on the public EEG emotion datasets SEED (Zheng and Lu, 2015) and DEAP (Koelstra et al., 2011).

The advantages of MBCC are as follows: (1) An effective discriminative dictionary is trained using the dictionary learning model framework by capturing common shared feature information from multi-frequency band data. The first correlation between data in multiple frequency bands is represented by the common shared dictionary. It creates a link between data from different frequency bands in order to obtain a new feature representation of EEG data. (2) Take into account the complementarity and difference of frequency band data, the projection matrix of each frequency band has the common shared and independent components. The common shared component reflects the second correlation between multiple frequency bands and can keep each frequency band consistent. (3) To assess the model’s discriminative ability, the Fisher criterion based on coding error is introduced in the projection space. Furthermore, the PCA-like regularization term based on the common shared projection component contributes to obtain more discriminative sparse coding.

## Background

Let **Z** = [**z**_1_,…,**z**_*n*_] ∈ **R**^*d*×*n*^ be a set of *d*-dimensional *n* training signals. The traditional dictionary learning is to learn a dictionary matrix to sparsely represent the EEG signals Z . The problem of dictionary learning (Jiang et al., 2013; Gu et al., 2021b) is,


(1) $$\begin{array}{c} \min_{D,A}{||\text{Z}-\text{DA}||}_{F}^{2}+\lambda \,{||\text{A}||}_{1},\\ s.t.\forall i,{||{\text{d}}_{i}||}_{0}=1, \end{array}$$

where *D* = [**d**_1_,…,**d**_*k*_] ∈ **R**^*d*×*K*^ is the learned dictionary, *K* is the dictionary size. A = [**a**_1_,…,**a**_*n*_] ∈ **R**^*K*×*n*^ is the sparse coding coefficient matrix. The first term in Eq. (1) is to minimize the reconstruction errors of **Z**. The second term is the sparsity constraints.

In our previous work OPFDDL method (Gu et al., 2021a), ${\text{Z}}^{m}=\left[{\text{z}}_{1}^{m},\ldots ,{\text{z}}_{n_{m}}^{m}\right]\in {\text{R}}^{d\times n_{m}}$is the class *m* frequent band signal set, *m* = 1,2,…,*M*, *n* = ∑_*m*_*n*_*m*_. By introducing the frequent band specific projection matrix **G**^*m*^ ∈ **R**^*d*×*p*^, each training signal ${\text{z}}_{j}^{m}$ is projected into a low-dimensional space, as ${\text{G}}^{m}\,{\text{z}}_{j}^{m}$. Suppose ${\text{S}}_{w}^{m}$ and ${\text{S}}_{b}^{m}$ are within-class and between-class reconstruction errors of the *m*-th frequent band signals, respectively. $S_{w}^{m}$ and $S_{b}^{m}$ are defined as,


(2) $$\begin{array}{c} {\text{S}}_{w}^{m}=Tr(\sum_{j=1}^{n_{m}}\left({\left({\text{G}}^{m}\right)}^{T}{\text{z}}_{j}^{m}-{\left({\text{G}}^{m}\right)}^{T}\text{D}\delta \left({\text{a}}_{j}^{m}\right)\right)\times ({\left({\text{G}}^{m}\right)}^{T}{\text{z}}_{j}^{m}\\ {-{\text{G}}^{m\,T}\text{D}\delta \left({\text{a}}_{j}^{m}\right))}^{T})\\ =T\,r\,\left({\text{G}}^{m\,T}\,{\text{W}}_{w}^{m}\,{\text{G}}^{m}\right) \end{array}$$

where ${\text{W}}_{w}^{m}=\sum_{j}^{n_{m}}\left({\text{z}}_{j}^{m}-\text{D}\,\delta \,\left({\text{a}}_{j}^{m}\right)\right)\times {\left({\text{z}}_{j}^{m}-\text{D}\,\delta \,\left({\text{a}}_{j}^{m}\right)\right)}^{T}$. The function $\delta \,\left({\text{a}}_{j}^{m}\right)$ returns the coding coefficients consistent with the class of ${\text{z}}_{j}^{m}$.


(3) $$\begin{array}{c} {\text{S}}_{b}^{m}=Tr(\sum_{j}^{n_{m}}\left({\left({\text{G}}^{m}\right)}^{T}{\text{z}}_{j}^{m}-{\left({\text{G}}^{m}\right)}^{T}\text{D}\xi \left({\text{a}}_{j}^{m}\right)\right)\times ({\left({\text{G}}^{m}\right)}^{T}{\text{x}}_{j}^{m}\\ {-{\text{G}}^{m\,T}\text{D}\xi \left({\text{a}}_{j}^{m}\right))}^{T})\\ =T\,r\,\left({\left({\text{G}}^{m}\right)}^{T}\,{\text{W}}_{b}^{m}\,{\text{G}}^{m}\right) \end{array}$$

where ${\text{W}}_{b}^{m}=\sum_{j}^{n_{m}}\left({\text{z}}_{j}^{m}-\text{D}\,\xi \,\left({\text{a}}_{j}^{m}\right)\right)\times {\left({\text{z}}_{j}^{m}-\text{D}\,\xi \,\left({\text{a}}_{j}^{m}\right)\right)}^{T}$. The function $\xi \,\left({\text{a}}_{j}^{m}\right)$ returns the coding coefficients not consistent with the class of ${\text{z}}_{j}^{m}$.

According to the classification rule of Fisher criterion (Peng et al., 2020; Zhang et al., 2021), the OPFDDL method proposes the discriminative model on *M* frequent bands in the projection space,


(4) $$\begin{array}{c} \min_{{\text{G}}^{m},\text{D}}\frac{\sum_{m}T\,r\,\left({\text{G}}^{m\,T}\,{\text{W}}_{w}^{m}\,{\text{G}}^{m}\right)}{\sum_{m}T\,r\,\left({\text{G}}^{m\,T}\,{\text{W}}_{b}^{m}\,{\text{G}}^{m}\right)},\\ s.t.{\left({\text{G}}^{m}\right)}^{T}\,\left({\text{G}}^{m}\right)=\text{I},m=1,2,\ldots ,M \end{array}$$

Then the matrices $\tilde{\text{G}}$, ${\tilde{\text{W}}}_{w}$, and ${\tilde{\text{W}}}_{b}$ are defined as $\tilde{\text{G}}=\left[{\text{G}}^{1},{\text{G}}^{2},\ldots ,{\text{G}}^{M}\right]$, ${\tilde{\text{W}}}_{w}=\left[\begin{array}{c} {\text{W}}_{w}^{1}\,\cdots \,0\\ \vdots \;\ddots \,\vdots \\ 0\;\cdots \,{\text{W}}_{w}^{M} \end{array}\right]$, and ${\tilde{\text{W}}}_{b}=\left[\begin{array}{c} {\text{W}}_{b}^{1}\,\cdots \,0\\ \vdots \;\ddots \,\vdots \\ 0\;\cdots \,{\text{W}}_{b}^{M} \end{array}\right]$. With these definitions, the objective function of OPFDDL has the following form,


(5) $$\begin{array}{c} \min_{\tilde{\text{G}},\text{D},\lambda }\lambda^{2}\,T\,r\,\left({\tilde{\text{G}}}^{T}\,{\tilde{\text{W}}}_{w}\,\tilde{\text{G}}\right)-\lambda \,T\,r\,\left({\tilde{\text{G}}}^{T}\,{\tilde{\text{W}}}_{b}\,\tilde{\text{G}}\right),\\ s.t.{\tilde{\text{G}}}^{T}\,\tilde{\text{G}}=\text{I}, \end{array}$$

where λ is the adaptive weight parameter.

The training procedure of OPFDDL is given by Algorithm 1.

**ALGORITHM 1:** The OPFDDL algorithm.

Repeat
1. Calculate the coding coefficients by solving the following problem:
$\underset{A}{\min}{\left\|{\tilde{G}}^{T}Z-DA\right\|}_{F}^{2}+\text{λ}{\left\|A\right\|}_{1}\;\;\;\;\;\;\;\;\;\;\;\;\;\;\;$	(6)
2. Calculate the projection matrix by solving the following problem:
$({\text{λ}}^{2}{\tilde{W}}_{w}^{}-\text{λ}{\tilde{W}}_{b}^{})\tilde{G}=\gamma \tilde{G}\;\;\;\;\;\;\;\;\;\;\;\;\;\;\;$	(7)
3. Calculate the dictionary **D** by:
$D=D-\frac{{\text{λ}}_{D}}{n}\sum_{k}\frac{\partial L({\tilde{Z}}_{k})}{\partial D},k=1,...,d\;\;\;\;\;\;\;\;\;\;\;\;\;\;\;$	(8)
$\frac{\partial L({\tilde{Z}}_{k})}{\partial D}=2\tilde{G}{\tilde{G}}^{T}D(\Lambda \Lambda^{T}+Η{Η}^{T})-2\tilde{G}{\tilde{G}}^{T}{\tilde{Z}}_{k}(\Lambda^{T}+{Η}^{T})\;\;\;\;\;\;\;\;\;\;\;\;\;\;\;$	(9)
$\Lambda =[\delta_{}^{1},\delta_{}^{2},...,\delta_{}^{M}],\;Η=[\zeta_{}^{1},\zeta_{}^{2},...,\zeta_{}^{M}]\;\;\;\;\;\;\;\;\;\;\;\;\;\;\;$	(10)
4. Calculate the adaptive weightλ by:
$\lambda =\frac{tr({\tilde{G}}^{T}{\tilde{W}}_{b}^{}\tilde{G})}{2tr({\tilde{G}}^{T}{\tilde{W}}_{w}^{}\tilde{G})}\;\;\;\;\;\;\;\;\;\;\;\;\;\;\;$	(11)
Until convergence

## Multi-Frequent Band Collaborative Eeg Emotion Classification Method Based on Optimal Projection and Shared Dictionary Learning

### Objective Function of MBCC

The OPFDDL method can be regarded as the baseline algorithm of MBCC. The primary distinction between the MBCC method and OPFDDL is that, although OPFDDL also employs a projection matrix to project each frequency band to the subspace, the correlation between projection matrices is weak. The common shared component defined in MBCC is a key part of multi-frequent band collaborative learning. In addition, according to the consistency principle, the PCA-like regularization term in the shared potential space further captures the discriminative information contained among multiple frequency bands. Thus, the MBCC method can balance discriminative knowledge and multi-frequent band correlation in the projection space.

We look for a projection matrix in the MBCC method to project the data from *d*-dimensional space to *p*-dimensional space. This study assumes that the projection matrix ***G***^*m*^ ∈ **R**^*d*×*p*^ for each frequency band has two parts: the shared component ***G***^*0*^ ∈ ***R***^*d*×*p*^, which is a common shared matrix that reflects the correlation between different frequency bands, and the band specific component ${\tilde{G}}^{m}\in R^{d\times p}$, which is the projection matrix for each frequency band. The matrix is equal to the sum of the shared component and the band specific component,


(12) $${\text{G}}^{m}=\left(1-\sigma \right)\,{\text{G}}^{0}+\sigma \,{\tilde{\text{G}}}^{m},$$

where σ ∈ [0,1]is the balance parameter. When σ = 1, the projection matrix **G**^*m*^ is degenerated into the band specific matrix ${\tilde{\text{G}}}^{m}$, which is equivalent to the projection matrix in the OPFDDL method. When σ = 0, the model only learns the common shared matrix.

The projection of the signal in each frequency band is represented as,


(13) $${\left({\text{G}}^{m}\right)}^{T}\,{\text{z}}_{j}^{m}={\left(\left(1-\sigma \right)\,{\text{G}}^{0}+\sigma \,{\tilde{\text{G}}}^{m}\right)}^{T}\,{\text{z}}_{j}^{m}.$$

The within-class reconstruction error of the *m*-th frequent band in the projected space can be represented as


(14) $$\begin{array}{c} J_{w}^{m}=Tr(\sum_{j}^{n_{m}}\sum_{k}^{p}[\left(1-\sigma \right){\text{G}}^{0}{\left(:,k\right)}^{T}\left({\text{z}}_{j}^{m}-\text{D}\delta \left({\text{a}}_{j}^{m}\right)\right)+\\ {\sigma {\tilde{\text{G}}}^{m}{\left(:,k\right)}^{T}\left({\text{z}}_{j}^{m}-\text{D}\delta \left({\text{a}}_{j}^{m}\right)\right)]}^{2})\\ =T\,r\,\left({\left(\left(1-\sigma \right)\,{\text{G}}^{0}+\sigma \,{\tilde{\text{G}}}^{m}\right)}^{T}\,{\text{W}}_{w}^{m}\,\left(\left(1-\sigma \right)\,{\text{G}}^{0}+\sigma \,{\tilde{\text{G}}}^{m}\right)\right). \end{array}$$

The between-class reconstruction error of the *m*-th frequent band in the projected subspace can be represented as


(15) $$\begin{array}{c} J_{b}^{m}=Tr(\sum_{j}^{n_{m}}\sum_{k}^{p}[\left(1-\sigma \right){\text{G}}^{0}{\left(:,k\right)}^{T}\left({\text{z}}_{j}^{m}-\text{D}\xi \left({\text{a}}_{j}^{m}\right)\right)+\\ {\sigma {\tilde{\text{G}}}^{m}{\left(:,k\right)}^{T}\left({\text{z}}_{j}^{m}-\text{D}\xi \left({\text{a}}_{j}^{m}\right)\right)]}^{2})\\ =T\,r\,\left({\left(\left(1-\sigma \right)\,{\text{G}}^{0}+\sigma \,{\tilde{\text{G}}}^{m}\right)}^{T}\,{\text{W}}_{b}^{m}\,\left(\left(1-\sigma \right)\,{\text{G}}^{0}+\sigma \,{\tilde{\text{G}}}^{m}\right)\right). \end{array}$$

Thus, the Fisher criterion of all frequent bands is written as,


(16) $$\min_{\text{D},{\text{G}}^{0},{\tilde{\text{G}}}^{m}}\frac{\sum_{m}T\,r\,\left({\left(\left(1-\sigma \right)\,{\text{G}}^{0}+\sigma \,{\tilde{\text{G}}}^{m}\right)}^{T}\,{\text{W}}_{w}^{m}\,\left(\left(1-\sigma \right)\,{\text{G}}^{0}+\sigma \,{\tilde{\text{G}}}^{m}\right)\right)}{\sum_{m}T\,r\,\left({\left(\left(1-\sigma \right)\,{\text{G}}^{0}+\sigma \,{\tilde{\text{G}}}^{m}\right)}^{T}\,{\text{W}}_{b}^{m}\,\left(\left(1-\sigma \right)\,{\text{G}}^{0}+\sigma \,{\tilde{\text{G}}}^{m}\right)\right)}.$$

Because different frequent band data describe the same object, there must be an internal connection between them. The model maximizes the commonality of multiple frequent band data in the shared projection space using the consistency principle. When projecting the data from multiple bands to the optimal subspace, we need to preserve the discriminative information available in the original space. To solve this problem, we use a PCA-like regularization term as follows,


(17) $$J\,\left({\text{G}}^{0}\right)=\min_{{\text{G}}^{0}}\sum_{m}{||{\text{Z}}^{m}-{\text{G}}^{0}\,{\left({\text{G}}^{0}\right)}^{T}\,{\text{Z}}^{m}||}_{F}^{2}.$$

Ignoring the constant terms in *J*(**G**^0^), Eq. (17) can be represented as,


(18) $$\begin{array}{c} J\,\left({\text{G}}^{0}\right)=-\min_{{\text{G}}^{0}}\sum_{m}T\,r\,\left(\left({\left({\text{G}}^{0}\right)}^{T}\,{\text{Z}}^{m}\right)\,{\left({\left({\text{G}}^{0}\right)}^{T}\,{\text{Z}}^{m}\right)}^{T}\right)\\ =-\min_{{\text{G}}^{0}}\sum_{m}T\,r\,\left({\left({\text{G}}^{0}\right)}^{T}\,{\text{Z}}^{m}\,{\left({\text{Z}}^{m}\right)}^{T}\,{\text{G}}^{0}\right). \end{array}$$

Let **Θ**^*m*^ = **Z**^*m*^(**Z**^*m*^)^*T*^, Eq. (18) can be written as,


(19) $$J\,\left({\text{G}}^{0}\right)=-\min_{{\text{G}}^{0}}\sum_{m}T\,r\,\left({\left({\text{G}}^{0}\right)}^{T}\,\Theta^{m}\,{\text{G}}^{0}\right).$$

Combined the Fisher criterion and PCA-like regularization term, the objective function of MBCC is,


(20) $$\begin{array}{c} \min_{{\text{G}}^{0},{\tilde{\text{G}}}^{m},\text{D}}\frac{\begin{array}{c} \sum_{m}Tr({\left(\left(1-\sigma \right){\text{G}}^{0}+\sigma {\tilde{\text{G}}}^{m}\right)}^{T}{\text{W}}_{w}^{m}(\left(1-\sigma \right){\text{G}}^{0}\\ +\sigma {\tilde{\text{G}}}^{m}))-\alpha \sum {}_{m}Tr({\left({\text{G}}^{0}\right)}^{T}\Theta^{m}{\text{G}}^{0}) \end{array}}{\begin{array}{c} \sum_{m}Tr({\left(\left(1-\sigma \right){\text{G}}^{0}+\sigma {\tilde{\text{G}}}^{m}\right)}^{T}\\ {\text{W}}_{b}^{m}\left(\left(1-\sigma \right){\text{G}}^{0}+\sigma {\tilde{\text{G}}}^{m}\right)) \end{array}},\\ s.t.\forall m,{\left(\left(1-\sigma \right)\,{\text{G}}^{0}+\sigma \,{\tilde{\text{G}}}^{m}\right)}^{T}\,\left(\left(1-\sigma \right)\,{\text{G}}^{0}+\sigma \,{\tilde{\text{G}}}^{m}\right)=\text{I}. \end{array}$$

The projection matrix is orthogonal and it will result in an efficient procedure for optimization. We can see that the dictionary learned in the MBCC method have the stronger discriminative ability.

Define G = [G^0^;G^1^;…,**G**^*M*^] ∈ **R**^(*M* + 1)*d*×*p*^,**Ω**^*m*^ = [(1−σ)I_*d*_,σI_*d*_,…,σI_*d*_] ∈ R^*d*×(*M* + 1)*d*^, **Δ**^*m*^ = [I_*d*_,0_*d*×*d*_,…,0_*d*×*d*_] ∈ R^*d*×(*M* + 1)*d*^, $\Lambda =\sum_{m}^{M}{\left(\Omega^{m}\right)}^{T}\,{\text{W}}_{w}^{m}\,\Omega^{m}$,$\Theta =\sum_{m}^{M}{\left(\Delta^{m}\right)}^{T}\,\Theta^{m}\,\Delta^{m}$, $\text{H}=\sum_{m}^{M}{\left(\Omega^{m}\right)}^{T}\,{\text{W}}_{b}^{m}\,\Omega^{m}$, Eq. (20) is equivalent to,


(21) $$\min_{G,D}\frac{T\,r\,\left({\text{G}}^{T}\,\Lambda \,\text{G}\right)-\alpha \,T\,r\,\left({\text{G}}^{T}\,\Theta \,\text{G}\right)}{T\,r\,\left({\text{G}}^{T}\,\text{HG}\right)},s.t.{\text{G}}^{T}\,\text{G}=\text{I}.$$

By combining the two terms on the numerator, we can get,


(22) $$\min_{G,D}\frac{T\,r\,\left({\text{G}}^{T}\,\left(\Lambda -\alpha \,\Theta \right)\,\text{G}\right)}{T\,r\,\left({\text{G}}^{T}\,\text{HG}\right)},s.t.{\text{G}}^{T}\,\text{G}=\text{I}.$$

### Optimization

It is not easy to directly solve the variables **G** and **D** in the objective function. Therefore, we will take the alternative iterative optimization scheme to decompose the original problem into two sub-optimization problems.

Update **G**. For the given dictionary **D**, there must be a minimum ρ, which satisfies the following formulation,


(23) $$\frac{T\,r\,\left({\text{G}}^{T}\,\left(\Lambda -\alpha \,\Theta \right)\,\text{G}\right)}{T\,r\,\left({\text{G}}^{T}\,\text{HG}\right)}\geq \rho ,$$

We have $F\,\left(\rho \right)=\min_{\text{G}}T\,r\,\left({\text{G}}^{T}\,\left(\Lambda -\alpha \,\Theta \right)\,\text{G}\right)-\rho \,T\,r\,\left({\text{G}}^{T}\,\text{HG}\right)$.

As a result, we can define the function of ρ by,


(24) $$T\,r\,\left({\text{G}}^{T}\,\left(\Lambda -\alpha \,\Theta \right)\,\text{G}\right)-\rho \,T\,r\,\left({\text{G}}^{T}\,\text{HG}\right)\geq 0,$$

According to Zhang et al. (2017), (1) *F*(ρ) is a decreasing function of ρ. (2) *F*(ρ) = 0 if ρ = ρ*. In addition, the minimum ρ always exists.

Then ρ can be updated by,


(25) $$\begin{array}{c} \rho =\rho +\lambda_{\rho }\,\frac{F\,\left(\rho \right)}{F^{\prime }\,\left(\rho \right)},\\ F^{\prime }\,\left(\rho \right)=-T\,r\,\left({\text{G}}^{T}\,\text{HG}\right), \end{array}$$

where λ_ρ_ is the learning rate.

With the fixed ρ and **D**, the objective function of G is,


(26) $$\begin{array}{c} \min_{\text{G}}T\,r\,\left({\text{G}}^{T}\,\left(\Lambda -\alpha \,\Theta -\rho \,\text{H}\right)\,\text{G}\right),\\ s.t.{\text{G}}^{T}\,\text{G}=\text{I}, \end{array}$$

The optimization of G can be solved by the following eigenvalue decomposition,


(27) (Λ-α⁢Θ-ρ⁢H)⁢G=γ⁢G.

The columns of the matrix G are the eigenvectors with respect to the first *p* minimum eigenvalues of Eq. (27).

Update **D**. With the fixed G, the objective function of **D** is,


(28) $$\min_{\text{D}}\frac{T\,r\,\left({\text{G}}^{T}\,\Lambda \,\text{G}\right)}{T\,r\,\left({\text{G}}^{T}\,\text{HG}\right)},$$

Let D = [**D**_1_,**D**_2_,…,**D**_*C*_] be the learned dictionary, and **D**_*j*_ is the *j*-th class sub-dictionary. The Eq. (28) can be re-written as,


(29) $$J\,\left({\text{D}}_{j}\right)=\min_{{\text{D}}_{j}}\sum_{j=1}^{C}\frac{T\,r\,\left({\text{G}}^{T}\,\Lambda_{j}\,\text{G}\right)}{T\,r\,\left({\text{G}}^{T}\,\text{HG}\right)},$$

where $\Lambda_{j}=\sum_{m=1}\sum_{j\neq s}^{c}\left({\text{Z}}_{j}^{m}-{\text{D}}_{s}\,\Gamma_{j,s}^{m}\right)\times {\left({\text{Z}}_{j}^{m}-{\text{D}}_{s}\,\Gamma_{j,s}^{m}\right)}^{T}$, $\text{H}=\sum_{m=1}\sum_{j=1}^{c}\left({\text{Z}}_{j}^{m}-{\text{D}}_{j}\,\Gamma_{j,j}^{m}\right)\times {\left({\text{Z}}_{j}^{m}-{\text{D}}_{j}\,\Gamma_{j,j}^{m}\right)}^{T}$. $\Gamma_{j,s}^{m}$ and $\Gamma_{j,j}^{m}$ are the coding coefficient matrices corresponding to classes *s* and *j* of the *m*-th frequent band, respectively, where *s≠j*.

**D**_*j*_ can be updated by gradient descent method, in which **D**_*j*_ is computed as,


(30) $$\begin{array}{c} {\text{D}}_{j}={\text{D}}_{j}+\eta \,\partial J\,\left({\text{D}}_{j}\right),\\ \partial J\,\left({\text{D}}_{j}\right)=\frac{\partial J\,\left({\text{D}}_{j}\right)}{\partial \Lambda_{j}}\,\frac{\partial \Lambda_{j}}{\partial {\text{D}}_{j}}+\frac{\partial J\,\left({\text{D}}_{j}\right)}{\partial \text{H}}\,\frac{\partial \text{H}}{\partial {\text{D}}_{j}}. \end{array}$$

There is no connection between **Λ**_*j*_ and D_*j*_, i.e., $\frac{\partial \Lambda_{j}}{\partial {\text{D}}_{j}}=0$. Therefore, we only need compute $\frac{\partial J\,\left({\text{D}}_{j}\right)}{\partial \text{H}}\,\frac{\partial \text{H}}{\partial {\text{D}}_{j}}$.


(31) $$\frac{\partial J\,\left({\text{D}}_{j}\right)}{\partial \text{H}}=\frac{-T\,r\,\left({\text{G}}^{T}\,\Lambda_{j}\,\text{G}\right)\,{\left(\text{G}\right)}^{T}\,\text{G}}{{\left(T\,r\,\left({\text{G}}^{T}\,\text{HG}\right)\right)}^{2}},$$

(32) $$\frac{\partial \text{H}}{\partial {\text{D}}_{j}}={\left(\Gamma_{j,j}^{m}\right)}^{T}\,\left({\text{D}}_{j}\,\Gamma_{j,j}^{m}-{\text{Z}}_{j}^{m}\right).$$

Update **A**. With the fixed **D** and **G**, the sparse coding coefficient matrix **A** can be computed as,


(33) $$\min_{\text{A}}{||{\text{G}}^{T}\,Z-\mathrm{DA}||}_{F}^{2}+\lambda \,{||\text{A}||}_{1},$$

Since **A** is differentiable, it can be obtained by,


(34) $$\text{A}={\left({\text{D}}^{T}\,\text{D}+\lambda \,\text{I}\right)}^{-1}\,\left({\text{D}}^{T}\,{\text{G}}^{T}\,\text{Z}\right).$$

The alternating optimization procedure of MBCC is summarized in Algorithm 2.

**ALGORITHM 2:** The MBCC algorithm.

Repeat
1. Calculate the coding coefficient matrix **A** by Eq. (34)
2. Calculate the projection matrix **G** by Eq. (27)
3. Calculate the dictionary **D** by Eq. (30)
Until convergence

### Testing

For the testing procedure, each frequency band feature of the signal z is represented as **z**^*m*^. With the obtained{**G**^*m*^,D}by Algorithm 2, its label *l*(*z*^*m*^) on the *m*-th frequency band can be computed by the following optimization problem,


(35) $$l\,\left({\text{z}}^{m}\right)=\min_{{\text{z}}^{m}}{||{\left({\text{Q}}^{m}\right)}^{T}\,{\text{z}}^{m}-{\text{D}}_{j}\,{\left({\text{D}}_{j}^{T}\,{\text{D}}_{j}\right)}^{-1}\,{\text{D}}_{j}^{T}\,{\text{z}}^{m}||}_{2}.$$

Then the majority voting strategy is used to determine the class label of signal z,


(36) $$y=\arg\,\max_{m}l\,\left({\text{z}}^{m}\right).$$

## Experiment

### Datasets and Experimental Settings

Two EEG emotion recognition data sets used in the experiment, SEED and DEAP datasets, which are described as follows. The SEED dataset is an emotional EEG dataset collected and provided by Shanghai Jiao Tong University’s BCMI Laboratory. The dataset is completed by requiring participants to wear EEG acquisition equipment and recording the emotional EEG signals produced by watching three different types of movie clips. Sixty-two channel electrodes are used in the SEED dataset. The dataset was compiled from 15 participants. With a total of 15 clips, the films are classified as positive, negative, or neutral in terms of their emotional impact. There are five clips of each type, and each movie clip lasts about 4 min. To ensure the experiment’s validity and accuracy, the playback sequence of the 15 videos is random, with no repeated clips. Every participant repeated the experiment three times. A few days were set aside in the middle of each experiment to allow participants to adjust their emotions so that they had a consistent emotional response to the same movie clip. In the experiment, EEG signals are divided into 5-s segments and features are extracted every 0.5 s. Thus, the sequence length of each segment is 19.

The DEAP dataset is another open database for emotion recognition research that uses EEG and peripheral physiological signals. The dataset recorded the EEG data and 13 peripheral physiological signals of 32 participants using music videos as stimulus materials. The DEAP dataset employs 40 music videos, each of which is 1 min long, as stimulus materials. These music videos are labeled and screened using the general three-dimensional model of valence, arousal, and dominance.

To illustrate the effectiveness of the MBCC method, the comparison methods in the experiment are: SVM (Cortes and Vapnik, 1995), LC-KSVD (Jiang et al., 2013), multi-view CVM (MvCVM) (Huang et al., 2016), global and local structural risk minimization (GLSRM) (Zhu et al., 2016), multi-view learning with universum (MVU) (Wang et al., 2014), and OPFDDL (Gu et al., 2021a). In detail, the Gaussian kernel is used in MvCVM. The kernel parameter and the weight parameter are searched in the grid {1/64, 1/32, …, 64} and {1, 10^1^, …, 10^3^}, respectively. The weights and offsets in GLSRM are searched in the grid{0.1, 0.2, …, 1}, and its regularization parameters are searched in the grid {1, 10^1^, …, 10^3^}. In MVU, the learning rate is 0.99, and the relaxation of views is 10^–6^. In OPFDDL and MBCC, the number of atoms in each class is selected in {5, 10, …, 35}. The dimension of matrix **G** is set to be 90% of the dimension of the EEG signal features. The parameter α is searched in the grid{0.1, 0.2, …, 1}. The parameter σ is set as σ = 1−α. The regularization parameter in Eq. (2) was set as 0.01. All methods are implemented in MATLAB.

### Experiments on the SEED Dataset

The commonly used power spectral density (PSD) features (Jenke et al., 2014) are adopted in δ, θ, α, β, and γ frequent bands. We obtain 62 dimensional features on each band. We divided the EEG signal data corresponding to the 15 movie clips collected and used 12 clips as training data and the remaining three clips as test data. In both the training and test sets, the proportion of three classes of EEG signals is the same. After the final preprocessing, the samples of three different classes of EEG signals in the training and test sets are balanced. The SEED dataset is divided into three sessions (sessions 1–3) according to the time interval of signal acquisition. The classification results of all methods in three sessions are shown in Tables 2–4. We can see that the MBCC method performs the best in terms of accuracy in all three sessions. In Table 2, the accuracies of the MBCC method are 0.67, 0.94, 1.26, and 1.39% better than the second best method OPFDDL in multi-frequent bands β + γ,α + β + γ,θ + α + β + γ,δ + θ + α + β + γ. The results in Tables 3, 4 are similar to those in Table 2. Compared with the OPFDDL method, the proposed MBCC has the ability to take into account the complementarity and consistency between frequency bands while maintaining the PCA constraints of the data structure in the projection space, which is conducive to improving classification performance. Thus, the dictionary learned in the projection space by MBCC has good discriminative performance. The SVM and LC-KSVD methods merge all frequency band data into a vector for learning, and they cannot effectively find the internal connection between each frequency band. For joint learning of multiple perspectives, MvCVM, GLSRM, and MVU treat each frequency band as a learning view. Obviously, the MBCC method obtains a more discriminative model based on dictionary learning and subspace learning.

**TABLE 2:** The accuracy (standard deviations) of all methods on SEED dataset in session 1.

Methods	β + γ	α + β + γ	θ + α + β + γ	δ + θ + α + β + γ
SVM	77.87	77.96	79.07	81.02
	(8.69)	(9.42)	(9.96)	(8.26)
LC-KSVD	78.39	80.10	81.65	83.50
	(9.81)	(8.13)	(9.19)	(8.38)
MvCVM	81.03	81.89	83.01	83.84
	(9.84)	(8.72)	(9.67)	(9.93)
GLSRM	81.62	82.84	83.33	84.17
	(9.66)	(8.95)	(8.11)	(9.39)
MVU	81.65	82.85	83.08	84.27
	(9.93)	(9.50)	(8.67)	(9.04)
OPFDDL	81.18	83.67	84.81	86.52
	(8.51)	(8.18)	(8.76)	(8.59)
MBCC	**81.85**	**84.61**	**86.07**	**87.91**
	(7.98)	(8.69)	(8.82)	(8.26)

*The best performance of each comparison is emphasized by the bold font.*

**TABLE 3:** The accuracy (standard deviations) of all methods on SEED dataset in session 2.

Methods	β + γ	α + β + γ	θ + α + β + γ	δ + θ + α + β + γ
SVM	77.25	78.04	79.67	80.92
	(8.53)	(7.81)	(7.96)	(9.92)
LC-KSVD	78.31	79.63	80.88	82.37
	(7.74)	(7.23)	(9.65)	(9.62)
MvCVM	80.53	82.55	83.37	83.94
	(8.28)	(8.33)	(8.24)	(9.54)
GLSRM	80.78	82.66	82.70	83.94
	(7.09)	(8.75)	(8.56)	(9.11)
MVU	81.76	82.51	82.84	84.24
	(8.88)	(8.89)	(9.57)	(9.82)
OPFDDL	81.30	83.22	84.76	86.21
	(8.75)	(8.78)	(9.90)	(9.43)
MBCC	**81.90**	**84.24**	**86.14**	**87.87**
	(8.52)	(7.99)	(8.85)	(8.07)

*The best performance of each comparison is emphasized by the bold font.*

**TABLE 4:** The accuracy (standard deviations) of all methods on SEED dataset in session 3.

Methods	β + γ	α + β + γ	θ + α + β + γ	δ + θ + α + β + γ
SVM	77.19	78.24	79.31	80.78
	(9.23)	(9.65)	(9.41)	(9.50)
LC-KSVD	77.61	79.76	80.12	81.92
	(8.12)	(7.09)	(7.32)	(9.87)
MvCVM	79.87	82.14	83.18	83.53
	(8.67)	(8.12)	(8.02)	(9.13)
GLSRM	80.45	81.30	82.43	83.28
	(9.09)	(9.59)	(9.34)	(8.84)
MVU	80.84	81.32	83.00	83.94
	(9.27)	(8.13)	(9.72)	(9.28)
OPFDDL	81.05	83.18	84.61	86.43
	(7.84)	(9.67)	(9.08)	(9.86)
MBCC	**81.81**	**84.24**	**85.63**	**87.74**
	(8.04)	(8.98)	(8.22)	(8.90)

*The best performance of each comparison is emphasized by the bold font.*

**FIGURE 1:**
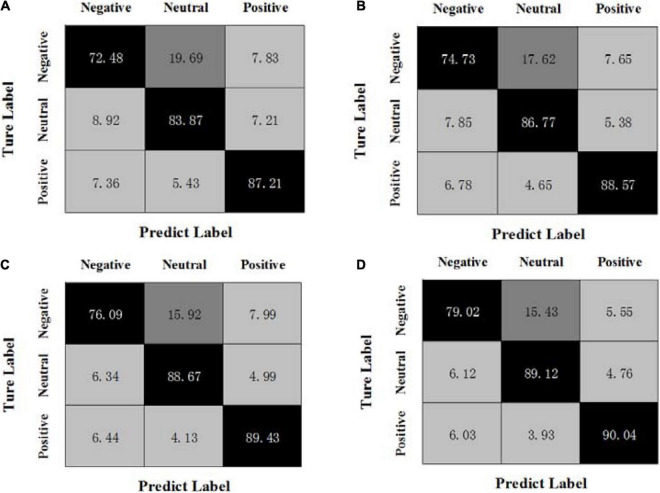
Confusion matrices of MBCC on the SEED dataset, **(A)** β + γ, **(B)** α + β + γ, **(C)** θ + α + β + γ, **(D)** δ + θ + α + β + γ.

**FIGURE 2:**
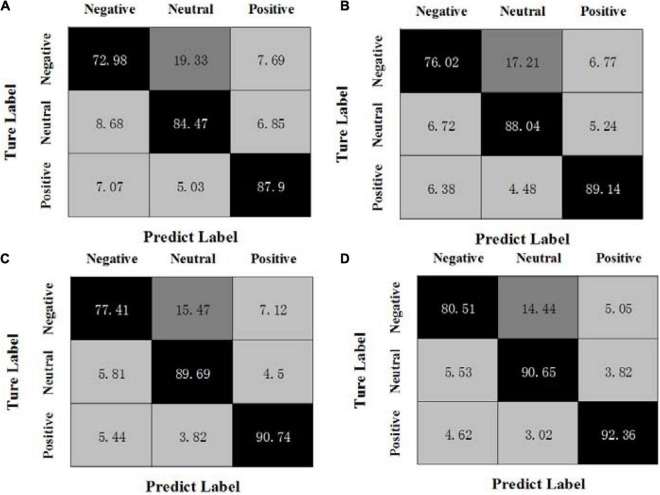
Confusion matrices of OPFDDL on the SEED dataset, **(A)** β + γ, **(B)** α + β + γ, **(C)** θ + α + β + γ, **(D)** δ + θ + α + β + γ.

By calculating the average results of all experiments on three sessions, Figures 1, 2 show the confusion matrices of MBCC and OPFDDL on the SEED dataset, respectively. The real label is represented by the ordinate of the confusion matrix, while the predicted label is represented by the abscissa.

It can be seen from Figures 1, 2 that (1) the classification results of positive emotional EEG signals are relatively good on the SEED dataset, while the classification results of negative emotions are relatively poor. Positive emotion is easier to identify than negative and neutral ones. This shows that positive emotions can cause similar brain feedback between frequency bands more than neutral and negative emotions. (2) The data of different frequency bands are projected into subspaces, and the common shared component of the projection matrix represents the correlation between frequency bands. Obviously, the OPFDDL method does not have this characteristic. (3) In addition, the MBCC method use the PCA-like regularization term based on shared projection matrix to make full use of the discriminative information of EEG data. Thus, the MBCC method achieves better classification accuracy on the SEED dataset.

### Experiments on the DEAP Dataset

In the DEAP dataset, music video stimulation is a three-dimensional emotion model based on valence, arousal, and dominance. The valence and arousal of emotion are classified in this subsection. The binary valence-oriented classification refers to the classification of emotions according to high valence and low valence. Also, the binary arousal-oriented classification refers to the classification of emotions according to high arousal and low arousal. The classification threshold is set to 5, the participant’s score ∈ [1,5]for valence is low valence, and score ∈ (5,9] is high valence. Similarly, the participant’s score ∈ [1,5]for arousal is low arousal, and score ∈ (5,9] is high arousal. The EEG signals are segmented by a 4-s time window with an overlap 2 s for each frequency band. Similar to the feature extraction strategy in subsection “Experiments on the SEED Dataset,” PSD features are used in the DEAP dataset. Following (Shen et al., 2021), four frequency bands (θ,α,β,γ) are used in the experiment.

Tables 5, 6 compare the average recognition results on valence and arousal on the DEAP dataset, respectively. We can see that (1) all methods have achieved the better classification accuracy for the arousal than the valence on the DEAP dataset. The reason may be that arousal, as an indicator of physiological arousal, reflects the degree of activation of neurophysiological activities, which can be directly reflected in changes in physiological signals. The valence-oriented classification is a more complex task involving mental state, and PSD features may not fully reflect valence’s state. (2) Compared with the benchmark methods SVM and LC-KSVD, the MBCC method has achieved much better results. Compared with GLSRM, MVU, and OPFDDL methods, the classification performance of the MBCC method has further improved. The MBCC method has the accuracy rate of 69.97% for the valence-oriented classification, and 71.55% for the arousal-oriented classification using four frequency bands. The classification accuracies of the MBCC method are increased by 0.89 and 0.96%, respectively, when compared to the second best method. This is due to that the multi-frequent band data maintains the consistency between feature similarity and semantic similarity in the learned subspace and can learn a more discriminative dictionary shared by frequency bands.

**TABLE 5:** The accuracy (standard deviations) of all methods on the DEAP dataset in valence.

Methods	β + γ	α + β + γ	θ + α + β + γ
SVM	62.21	63.04	63.55
	(8.30)	(8.09)	(8.76)
KSVD	62.63	63.51	63.94
	(8.59)	(8.28)	(8.43)
MvCVM	63.87	64.20	64.65
	(8.57)	(8.42)	(9.07)
GLSRM	64.15	66.26	66.84
	(9.60)	(8.71)	(9.35)
MVU	64.14	66.18	66.79
	(9.02)	(9.29)	(9.13)
OPFDDL	66.04	68.42	69.08
	(8.74)	(8.40)	(8.95)
MBCC	**66.64**	**68.85**	**69.97**
	(8.65)	(8.20)	(8.46)

*The best performance of each comparison is emphasized by the bold font.*

**TABLE 6:** The accuracy (standard deviations) of all methods on the DEAP dataset in arousal.

Methods	β + γ	α + β + γ	θ + α + β + γ
SVM	64.77	65.37	65.85
	(10.85)	(11.67)	(10.94)
KSVD	65.07	66.07	66.20
	(10.46)	(11.09)	(11.86)
MvCVM	66.31	66.90	67.19
	(11.01)	(11.48)	(11.24)
GLSRM	66.49	69.05	69.49
	(10.33)	(10.27)	(10.48)
MVU	66.38	69.10	69.27
	(10.79)	(10.75)	(11.12)
OPFDDL	68.46	70.35	70.59
	(10.56)	(10.87)	(10.06)
MBCC	**69.14**	**70.96**	**71.55**
	(10.39)	(10.88)	(10.70)

*The best performance of each comparison is emphasized by the bold font.*

**FIGURE 3:**
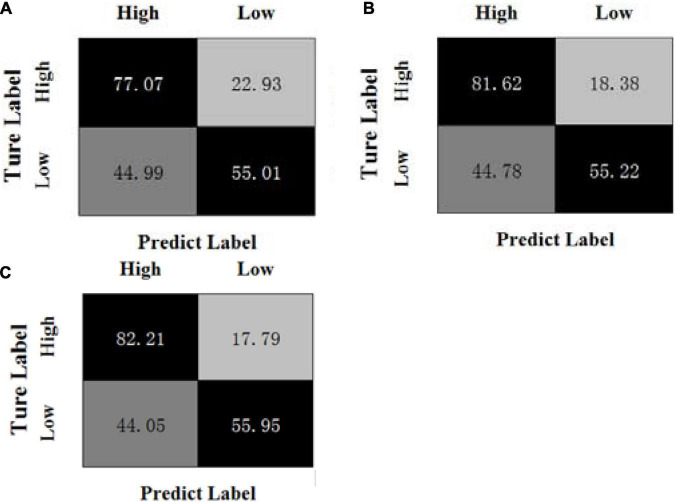
Confusion matrices of OPFDDL on the DEAP dataset in valence, **(A)** β + γ, **(B)** α + β + γ, **(C)** θ + α + β + γ.

**FIGURE 4:**
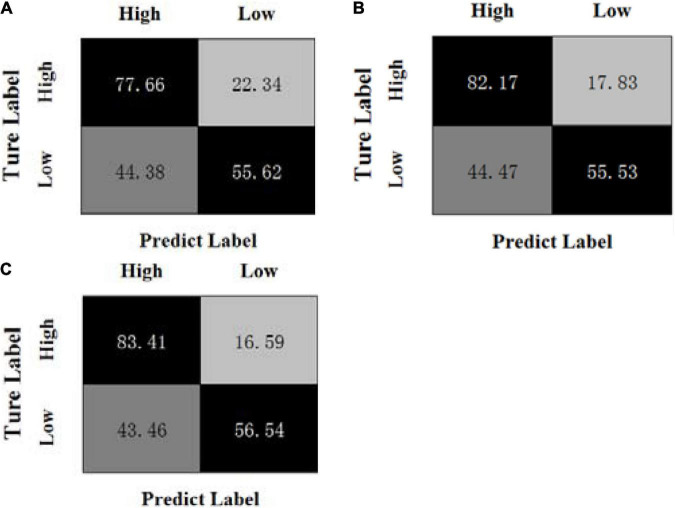
Confusion matrices of MBCC on the DEAP dataset in valence, **(A)** β + γ, **(B)** α + β + γ, **(C)** θ + α + β + γ.

**FIGURE 5:**
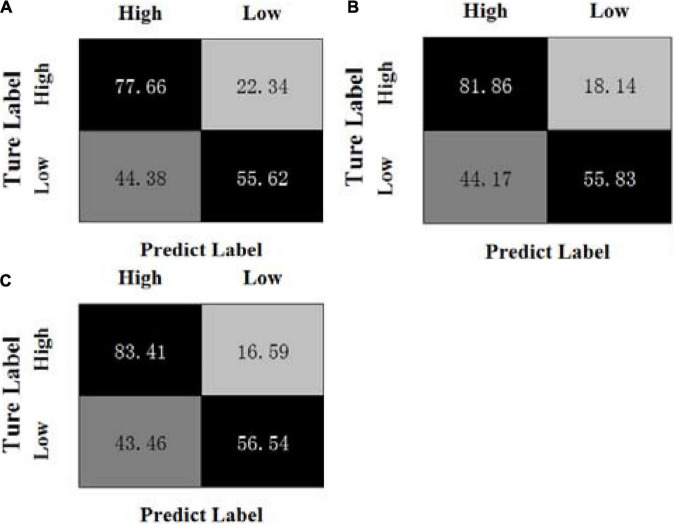
Confusion matrices of OPFDDL on the DEAP dataset in arousal, **(A) β + γ**, **(B) α + β + γ**, **(C) θ + α + β + γ**.

**FIGURE 6:**
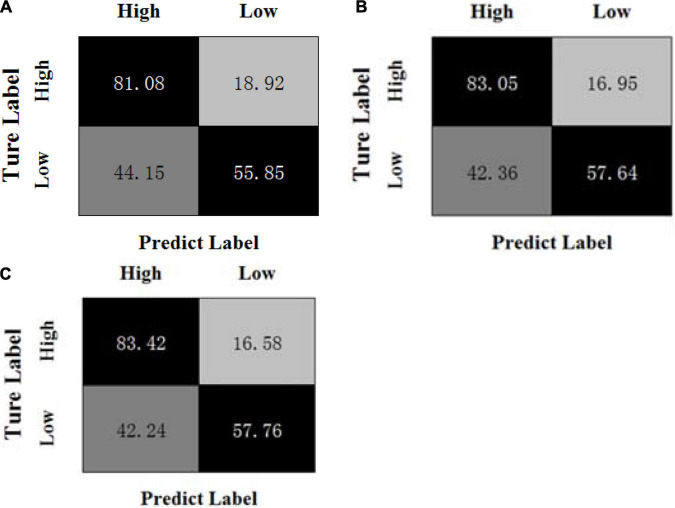
Confusion matrices of MBCC on the DEAP dataset in arousal, **(A) β + γ**, **(B) α + β + γ**, **(C) θ + α + β + γ**.

Figures 3, 4 show the confusion matrices of the OPFDDL method and the MBCC method in valence, respectively. Figures 5, 6 show the confusion matrices of the OPFDDL method and the MBCC method in arousal, respectively. Compared with OPFDDL, MBCC has obvious advantages in valence-oriented and arousal-oriented classifications. When different band data describe the same object, there must be an internal connection between each band data. According to the consistency principle, the MBCC method maximizes the commonness of multiple frequent bands in the shared projection space. Furthermore, the Fisher criterion and PCA-like regularization term aids in learning more discriminative sparse representation and maintaining data structure.

### Parameter Analysis

**FIGURE 7:**
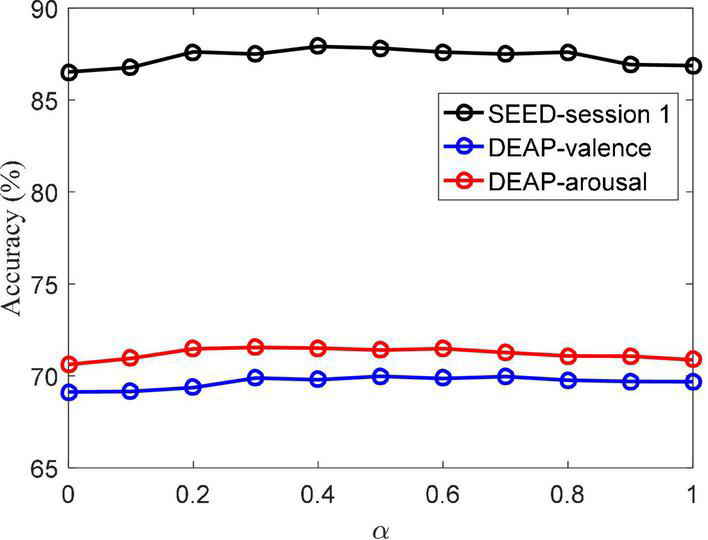
Classification accuracy of MBCC vs. different α on the SEED and DEAP datasets.

The parameter involved in the objective function of the MBCC method is α, and its impact on MBCC’s performance is analyzed here. The set value range specifies how to conduct experiments on the SEED session 1 and DEAP dataset, respectively. Figure 7 depicts the accuracy values at various values of α. The figure shows that MBCC achieves the highest accuracy value when taking 0.4, 0.5, and 0.6 on the SEED session 1, DEAP in valence, and DEAP in arousal, respectively.

**FIGURE 8:**
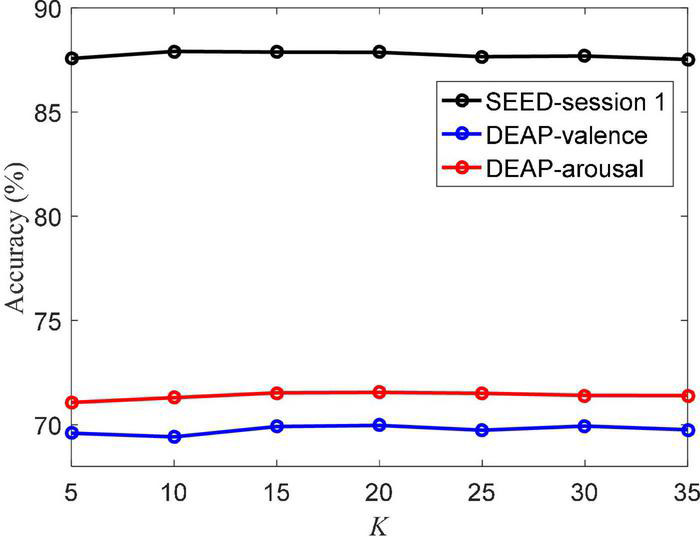
Classification accuracy of MBCC vs. different *K* on the SEED and DEAP datasets.

The atomic number *K* of the dictionary also directly determines the performance of the MBCC method. Figure 8 shows the accuracy values under different *K* values. We can see that when *K* reaches 15 and 20 on the SEED session 1 and DEAP dataset, respectively, the accuracy rate tends to stabilize. It indicates that the learned dictionary well represents the data characteristics of the EEG data. Also it shows that the MBCC method can be well applied to the SEED and DEAP datasets using a small size of dictionary.

## Conclusion

According to the consistent complementarity of multi-frequent band EEG signals and the internal correlation of data itself, this study proposes multi-frequency band collaborative EEG emotion classification method based on the idea of dictionary learning and subspace learning. Using a projection matrix, this method maps different frequency band data to the subspaces of the same dimension. Unlike most existing projection strategies, the projection matrix we designed is divided into two parts, a common shared component and a band-specific component. This strategy can fully use the relevance of different frequency bands and their shared semantics. In the subspace, the MBCC method learns the common shared dictionary between the frequency bands, which can represent the correlation and discrimination of the EEG data. Simultaneously, the incorporation of Fisher criterion and PCA-like regularization term into the subspace *via* dictionary learning makes the learned model more discriminative.

However, the time computation of MBCC is relatively high. It may be not suitable for real-time predicting emotional states in applications of human-computer interaction. This is the problem we need to solve in the next stage. Furthermore, the work that can be studied further in the future includes: (1) Human emotions are susceptible to external influences. For example, the emotions of the subjects may change while watching a film. The overall emotions of watching the film may be consistent, but the emotions may be inconsistent with expectations at times. As a result, the collected EEG signals are mixed with abnormal samples. In practice, selecting the appropriate abnormal sample processing method is important. The use of the correct processing method can improve the accuracy of emotional EEG signal recognition. (2) EEG signals have the characteristics of randomness. That is, for the same individual subjects, EEG signals are different even in the same emotional state at different times. How to improve the robustness of emotion classification model in multiple domains still needs further research. In the future, we will continue to design and improve our method to be suitable in across time and individuals scenes.

## Data Availability Statement

Publicly available datasets were analyzed in this study. The SEED and DEAP datasets analyzed for this study can be found in the following links, respectively: http://bcmi.sjtu.edu.cn/s̃eed/seed.html, http://www.eecs.qmul.ac.uk/mmv/datasets/deap/.

## Author Contributions

JZ and TN conceived and designed the proposed model. ZS performed the experiment. All authors drafted and approved the manuscript.

## Conflict of Interest

The authors declare that the research was conducted in the absence of any commercial or financial relationships that could be construed as a potential conflict of interest.

## Publisher’s Note

All claims expressed in this article are solely those of the authors and do not necessarily represent those of their affiliated organizations, or those of the publisher, the editors and the reviewers. Any product that may be evaluated in this article, or claim that may be made by its manufacturer, is not guaranteed or endorsed by the publisher.

## References

[B1] Aranha R. V. Corrêa C. G. Nunes F. (2019). Adapting software with affective computing: a systematic review. *IEEE Trans. Affect. Comp.* 12 883–899. 10.1109/TAFFC.2019.2902379

[B2] Cortes C. Vapnik V. (1995). Support vector machine. *Mach. Learn*. 20 273–297. 10.1007/BF00994018

[B3] Davis M. Whalen P. (2001). The amygdala: vigilance and emotion. *Mol. Psychiatry* 6 13–34. 10.1038/sj.mp.4000812 11244481

[B4] Gianaros P. J. Marsland A. L. Kuan C. H. Schirda B. L. Jennings J. R. Sheu L. K. (2014). An inflammatory pathway links atherosclerotic cardiovascular disease risk to neural activity evoked by the cognitive regulation of emotion. *Biol. Psychiatry* 75 738–745. 10.1016/j.biopsych.2013.10.012 24267410 PMC3989430

[B5] Gu X. Fan Y. Q. Zhou J. Zhu J. Q. (2021a). Optimized projection and fisher discriminative dictionary learning for eeg emotion recognition. *Front. Psychol.* 12:705528. 10.3389/fpsyg.2021.705528 34262515 PMC8274488

[B6] Gu X. Zhang C. Ni T. (2021b). A hierarchical discriminative sparse representation classifier for EEG signal detection. *IEEE/ACM Trans. Comp. Biol. Bioinform.* 18 1679–1687. 10.1109/TCBB.2020.3006699 32750882

[B7] Guil R. Ruiz-González P. Merchán-Clavellino A. Morales-Sánchez L. Zayas A. Gomez-Molinero R. (2020). Breast cancer and resilience: the controversial role of perceived emotional intelligence. *Front. Psychol.* 11:595713. 10.3389/fpsyg.2020.595713 33384644 PMC7769870

[B8] Huang C. Chung F. Wang S. (2016). Multi-view L2-SVM and its multi-view core vector machine. *Neural Networks* 75 110–125. 10.1016/j.neunet.2015.12.004 26773824

[B9] Jenke R. Peer A. Buss M. (2014). Feature extraction and selection for emotion recognition from EEG. *IEEE Trans. Affect. Comput*. 5 327–339. 10.1109/TAFFC.2014.2339834

[B10] Jiang Z. Lin Z. Davis L. (2013). Label consistent K-SVD: learning a discriminative dictionary for recognition. *IEEE Trans. Patt. Anal. Mach. Intell*. 35 2651–2664. 10.1109/TPAMI.2013.88 24051726

[B11] Klatzkin R. R. Nolan L. J. Kissileff H. R. (2021). Self-reported emotional eaters consume more food under stress if they experience heightened stress reactivity and emotional relief from stress upon eating. *Physiol. Behav.* 243:113638. 10.1016/j.physbeh.2021.113638 34742909 PMC8717738

[B12] Koelstra S. Muhl C. Soleymani M. Lee J. S. Yazdani A. Ebrahimi T. (2011). Deap: a database for emotion analysis; using physiological signals. *IEEE Trans. Affect. Comput*. 3 18–31. 10.1109/T-AFFC.2011.15

[B13] Li J. Zhang Z. He H. (2018). Hierarchical convolutional neural networks for EEG-based emotion recognition. *Cogn. Comp.* 10 368–380. 10.1007/s12559-017-9533-x

[B14] Li M. Lu B. L. (2009). “Emotion classification based on gamma-band EEG”. *2009 Annual International Conference of the IEEE Engineering in Medicine and Biology Society*, Minneapolis, MN. 1223–1226. 10.1109/IEMBS.2009.5334139 19964505

[B15] Li W. Zhang Z. Song A. (2020). Physiological-signal-based emotion recognition: an odyssey from methodology to philosophy. *Measurement* 172:108747.

[B16] Li Y. Zheng W. Cui Z. Zong Y. Ge S. (2019). EEG emotion recognition based on graph regularized sparse linear regression. *Neural Proc. Lett.* 49 555–571. 10.1007/s11063-018-9829-1

[B17] Liu Y. Ding Y. Li C. Cheng J. Cheng J. Song R. C. (2020). Multi-channel EEG-based emotion recognition *via* a multi-level features guided capsule network. *Comp. Biol. Med.* 123:103927. 10.1016/j.compbiomed.2020.103927 32768036

[B18] Mohammadi Z. Frounchi J. Amiri M. (2017). Wavelet-based emotion recognition system using EEG signal. *Neural Comp. Appl.* 28 1985–1990. 10.1007/s00521-015-2149-8

[B19] Ni T. G. Ni Y. Y. Xue J. Wang S. H. (2021). A domain adaptation sparse representation classifier for cross-domain Electroencephalogram-based emotion classification. *Front. Psychol.* 12:721266. 10.3389/fpsyg.2021.721266 34393958 PMC8358659

[B20] Nurillaeva N. M. Abdumalikova F. B. (2021). Predictive importance of psycho-emotional syndrome of patients with coronary heart disease in the violation of platelet hemostatic system. *Atherosclerosis* 331:E204. 10.1016/j.atherosclerosis.2021.06.625

[B21] Peng Y. Liu S. Wang X. Wu X. (2020). Joint local constraint and fisher discrimination based dictionary learning for image classification. *Neurocomputing* 398 505–519. 10.1016/j.neucom.2019.05.103

[B22] Samsonovich A. V. (2020). Socially emotional brain-inspired cognitive architecture framework for artificial intelligence. *Cogn. Syst. Res.* 60 57–76. 10.1016/j.cogsys.2019.12.002

[B23] Shareef M. A. Mukerji B. Alryalat M. Wright A. Dwivedi Y. K. (2018). Advertisements on Facebook: identifying the persuasive elements in the development of positive attitudes in consumers. *J. Retail. Cons. Serv.* 43 258–268. 10.1016/j.jretconser.2018.04.006

[B24] Shen F. Peng Y. Kong W. Dai G. (2021). Multi-scale frequency bands ensemble learning for EEG-based emotion recognition. *Sensors* 21:1262. 10.3390/s21041262 33578835 PMC7916620

[B25] Tennant C. McLean L. (2001). The impact of emotions on coronary heart disease risk. *J. Cardiovas. Risk* 8 175–183. 10.1177/174182670100800309 11455850

[B26] Wang Z. Zhu Y. Liu W. Chen Z. Gao D. (2014). Multi-view learning with Universum. *Knowledge-Based Syst.* 70 376–391. 10.1016/j.knosys.2014.07.019

[B27] Wirkner J. Weymar M. Lw A. Hamm C. Anne-Marie S. Kirschbaum C. (2017). Cognitive functioning and emotion processing in breast cancer survivors and controls: an ERP pilot study. *Psychophysiology* 54 1209–1222. 10.1111/psyp.12874 28432781

[B28] Yang Y. Wu Q. Fu Y. Chen X. (2018). “Continuous Convolutional Neural Network with 3D Input for EEG-Based Emotion Recognition,” in *International Conference on Neural Information Processing*, Siem Reap, 433–443. 10.1007/978-3-030-04239-4_39

[B29] Zhang G. Sun H. Porikli F. Liu Y. Sun Q. (2017). Optimal couple projections for domain adaptive sparse representation-based classification. *IEEE Trans. Image Process*. 26 5922–5935. 10.1109/TIP.2017.2745684 28858805

[B30] Zhang G. Yang J. Zheng Y. Luo Z. Zhang J. (2021). Optimal discriminative feature and dictionary learning for image set classification. *Inform. Sci.* 547 498–513. 10.1016/j.ins.2020.08.066

[B31] Zhao G. Zhang Y. Yan G. (2018). Frontal EEG asymmetry and middle line power difference in discrete emotions. *Front. Behav. Neurosci.* 11:225. 10.3389/fnbeh.2018.00225 30443208 PMC6221898

[B32] Zheng W. L. Lu B. L. (2015). Investigating critical frequency bands and channels for EEG-based emotion recognition with deep neural networks. *IEEE Trans. Auton. Mental Dev* 7 162–175. 10.1109/tamd.2015.2431497

[B33] Zhong P. Wang D. Miao C. (2020). EEG-Based emotion recognition using regularized graph neural networks. *IEEE Trans. Affect. Comp. Early Access* 2020:2994159. 10.1109/TAFFC.2020.2994159

[B34] Zhu C. Wang Z. Gao D. (2016). New design goal of a classifier: global and local structural risk minimization. *Knowledge-Based Syst.* 100 25–49. 10.1016/j.knosys.2016.02.002

